# High Altitude Is Beneficial for Antioxidant Components and Sweetness Accumulation of Rabbiteye Blueberry

**DOI:** 10.3389/fpls.2020.573531

**Published:** 2020-09-25

**Authors:** Qilong Zeng, Gangqiang Dong, Liangliang Tian, Han Wu, Yongjun Ren, Guy Tamir, Wuyang Huang, Hong Yu

**Affiliations:** ^1^ Institute of Botany, Jiangsu Province and Chinese Academy of Sciences, Nanjing, China; ^2^ Amway (China) Botanical R&D Center, Wuxi, China; ^3^ Institute of Farm Product Processing, Jiangsu Academy of Agricultural Sciences, Nanjing, China; ^4^ School of Computer and Software, Nanjing University of Information Science & Technology, Nanjing, China; ^5^ Agricultural Research and Development, Central Mountain Region, Tekoa, Israel

**Keywords:** *Vaccinium ashei*, antioxidant compounds, anthocyanin, electronic tongue, sensory properties

## Abstract

To better understand the effect of growing location on the phytochemical compounds and sensory properties of blueberry (*Vaccinium* spp.), here we investigated rabbiteye blueberry ‘Brightwell’ (*Vaccinium ashei* cv. ‘Brightwell’) grown in 10 locations of China. Significant differences in terms of total soluble solids, titratable acidity, flavonoids, phenols, as well as proanthocyanidins and anthocyanins, were found in the fruits (berries) of blueberry plants among the different sampled locations. Furthermore, their sensory properties, which evaluated by the electronic tongue method, also significantly differed among the 10 locations. The content of flavonoids, phenols, proanthocyanidins, and anthocyanins all had significant correlations with sensory properties, except that of aftertaste-astringency. A key finding to emerge was that blueberry plants grown at high altitude locations harbored a high content of total soluble solids, flavonoids, phenols, proanthocyanidins, and anthocyanins along with high scores for the sweetness. These results suggested cultivating blueberry at high altitude can produce fruit that not only possess pronounced beneficial health effects but also good taste.

## Introduction

The cultivation of blueberry (*Vaccinium* spp.) in China has garnered dramatically rising interest over the last 15 years, with its production area having reached 22,000 hm^2^, or 16.3% that of world’s total (Li et al., 2016; Brazelton et al., 2017). Meanwhile, global blueberry acreage is continuing to increase due to their nutritional and health benefits (Scalzo et al., 2013; Upadhaya and Dwivedi, 2019).

Blueberry is a richer source of antioxidant compounds (flavonoid, polyphenolic compounds, and especially anthocyanins) when compared to other berries, such as strawberry or raspberry (Cardeñosa et al., 2016). Blueberry cultivars high in antioxidant compounds could command a price premium when marketing them to consumers (Gilbert et al., 2015; Kraujalytė et al., 2015; Gallardo et al., 2018). Furthermore, the sensory quality of the harvested berries is another important aspect influencing the behavior of their would-be consumers. Consumer preference studies have suggested that key sensory properties, such as sweetness, freshness, and juiciness, were another main psychological drive for purchasing blueberries (Saftner et al., 2008; Gilbert et al., 2014; Yue and Wang, 2016).

To better accommodate consumer preferences, hundreds of blueberry cultivars have been bred by scientists worldwide. Among them, phytochemical compounds were found to vary widely (Connor et al., 2002; Gilbert et al., 2014). Similarly, there exists much variation in the sensory scores for intensity of blue color, juiciness, sweetness, and blueberry-like flavor among different blueberry cultivars currently on the market (Saftner et al., 2008).

Phytochemical compounds in blueberry fruits were also considerably affected by the plant’s growing locations and the interaction between environment and cultivars, although contradictory results have been reported (Zoratti et al., 2015; Correia et al., 2016). Sensory properties are induced by a variety of phytochemical compounds, such as sugars, acids, polyphenol composition, total soluble solids (TSS), titratable acidity (TA), and other quality measurements related with sweetness, sourness, and blueberry-like flavor (Saftner et al., 2008; Barrett et al., 2010; Bett-Garber et al., 2015). Therefore, it is reasonable to speculate that blueberry’s sensory properties may also be influence by growing location. Yet, surprisingly, very little information is available on the effect of growing location upon blueberry sensory properties, and their correlation with phytochemical compounds (Saftner et al., 2008; Bett-Garber et al., 2015).

Traditionally, the taste of blueberries has been evaluated using human sensory panels, which provides an integrated, direct measurement of perceived intensities of target taste attributes (Hanson et al., 1993; Trigo et al., 2004; Moreno et al., 2007). However, this approach is quite time-consuming and expensive and it is confined to certain conditions (Liang et al., 2003; Liu et al., 2017). Electronic tongue (“E-tongue”) systems have been successfully developed to mimic the organization of human taste buds, which are sensor arrays (Wei and Wang, 2013; Phat et al., 2016). This instrument is widely used for testing liquid foodstuffs, such as water (Carbó et al., 2018), wine (Buratti et al., 2007), fruit juice (Qiu et al., 2015), and tea (Chen et al., 2008). It has proven to be a promising, rapid, and low-cost technique for the discrimination of blueberry juices produced from different plant genotypes (Kraujalytė et al., 2015). Accordingly, in this study, sensory taste variability of blueberry from different locations in China were evaluated using the E-tongue, with the aim of exploring the effect of growing location on blueberry sensory properties, and to clarify the relationship between sensory properties and phytochemical compounds in blueberry. Knowledge gained in our study may provide a useful guide for selecting orchard locations with a view towards improving fruit berry quality.

## Materials and Methods

### Plant Materials

**Figure 1:**
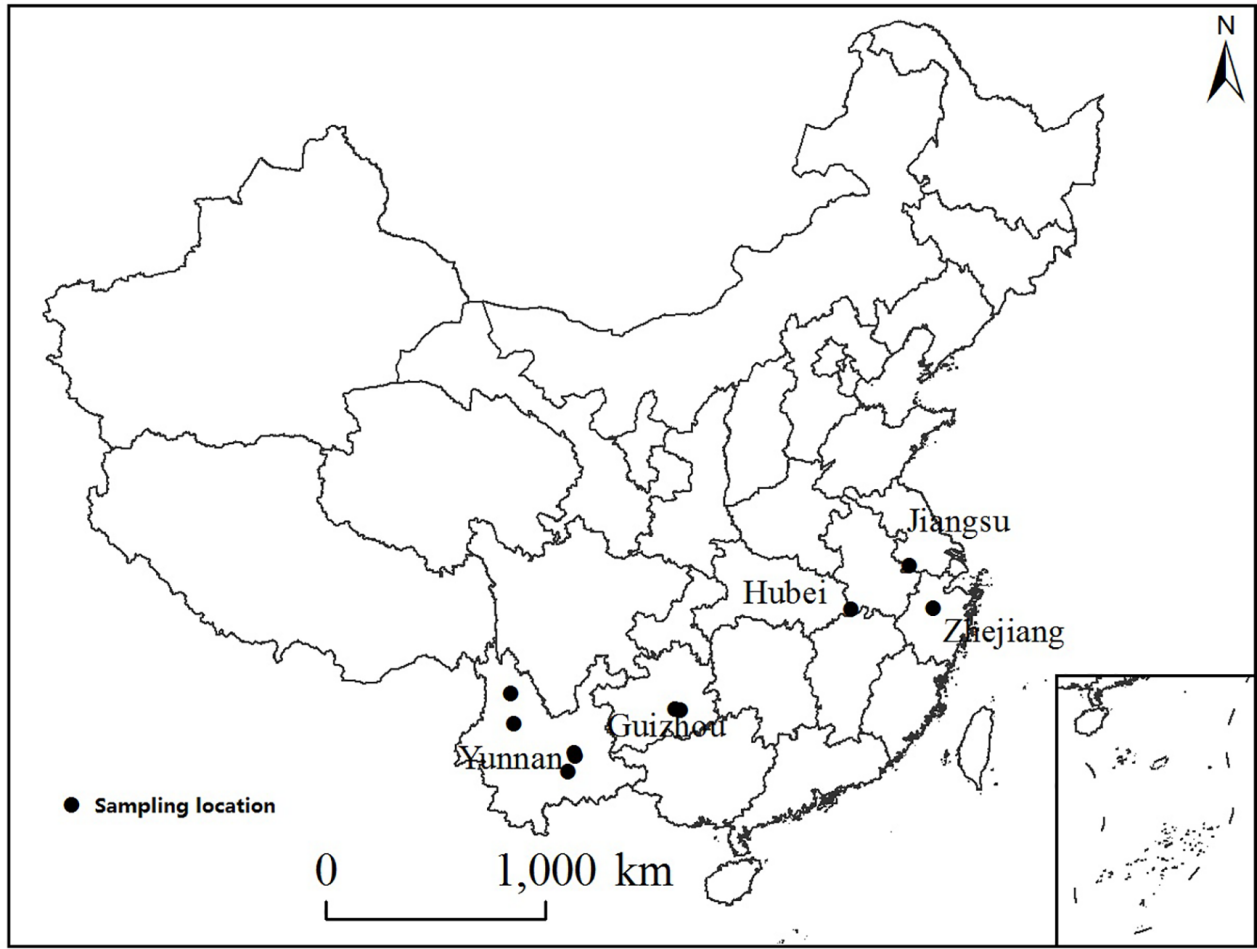
The locations of 10 fields sampled in five provinces of China.

Rabbiteye blueberry (*Vaccinium ashei* cv. ‘Brightwell’) fruits were sampled from five major blueberry-growing provinces of China, in July 2017 (**Figure 1**). Ten blueberry orchards known for their abundant yields were selected as sampling sites, whose location and altitude are listed in **Table 1**.

**Table 1:** Geographical details for the 10 sampled sites of blueberry in China.

Location		Abbreviation	Latitude (N)	Longitude (E)	Altitude (m)
Caota town, Zhejiang province	CT		29° 38’ 30”	120° 1’ 8”	199
Baima town, Jiangsu province	BM		31° 30’ 20”	119° 8’ 51”	49
Dushan town, Hubei province	DS		30° 2’ 24”	116° 4’ 21”	37
Xuanwei town, Guizhou province	XW		26° 22’ 56”	107° 44’ 24”	750
Xianchang town, Guizhou province	XC		26° 25’ 21”	107° 31’ 54”	912
Shigu town, Yunnan province	SG		26° 59’ 40”	99° 56’ 30”	1,950
Yinqiao town, Yunnan province	YQ		25° 45’ 28”	100° 7’ 13”	2,010
Yousuo town, Yunnan province	YS		24° 38’ 21”	102° 55’ 51”	1,731
Haikou town, Yunnan province	HK		24° 31’ 28”	102° 56’ 52”	1,781
Longpeng town, Yunan province	LP		23° 51’ 24”	102° 39’ 58”	1,840

Blueberries, that are the fruits, were manually picked at the commercial ripening stage, when the berries were fully developed in size and blue in color. Five plants (replicates) were randomly chosen in each orchard, and with, approximately, 120 g of blueberries randomly collected from each plant. These samples of harvested blueberries were then pooled and stored in a sealed foam box with ice to prevent their degradation while moving them from field to the laboratory, where they were stored at –20°C. All metabolic analyses were performed within 3 months from this field collection.

### Chemicals and Reagents

Folin–Ciocalteu reagent, catechin, and cyanidin 3-glucoside were purchased from Sigma-Aldrich (St. Louis, MO, USA), while rutin and gallic acid were purchased from J&K chemicals Ltd (Beijing, China). The HPLC solvents were bought from EMD (Darmstadt, Germany). Other chemicals and reagents used in this study were of analytical grade and were obtained from Sinopharm Chemical Reagent Co., Ltd. (Shanghai, China).

### Sample Preparation and Extraction

All analytical determinations were applied to a fruit homogenate, obtained by crushing about 50 g of frozen berries in a food blender (Philip, Shanghai, China). A portion of each sample’s homogenate was used to determine TSS content, TA, and sensory properties; the other part was used to extract phenols, flavonoids, proanthocyanidin, and anthocyanin

The extraction method was adapted from Wang et al. (2015), with some modifications. Approximately 1 g of each homogenized blueberry sample was put into a 50 ml centrifuge tube, in which it was mixed with 15 ml of 85% methanol containing 0.5% formic acid, and then shaken in a vortexer for 15 s to ensure even mixing. Each sample was sonicated at 20°C, for 20 min, in a KQ-5200E ultrasonic cleaner (Shumei, Kunshan, China), and then centrifuged at 5,000 rpm for 10 min. The supernatant was removed, and another 15 ml of the methanol extraction solution was added to the residue. This extraction operation was repeated three times per sample, until the berries had become colorless. These extracts were combined and stored at –20°C for later chemical analyses.

### Total Soluble Solid (TSS) and Titritable Acid (TA) Content

The TSS of berries was measured with a hand refractometer (Co. Atago, Japan) on the juice obtained by squeezing the homogenized blueberries. To determine TA, a 2.5 g sample of homogenized blueberries was first diluted with 25 ml of distilled water, after which TA was quantified with a neutralizing 0.1 M NaOH solution to an end-point of pH 8.1. Values of TA were expressed as the percent citric acid (mass/mass) on a fresh weight (FW) basis of fruit; the ratio between TSS and TA ratio was calculated as a taste indicator.

### Total Phenolic Content

Total phenolic content (TP) was estimated using the Folin–Ciocalteu colorimetric method, as described by Li et al. (2013). Briefly, appropriate dilutions of the extracted samples (0.2 ml) were oxidized with 2 ml of 0.5 mol L^-1^ Folin–Ciocalteu reagent, for 4 min, at room temperature. Then, the reactive solution was neutralized, by 2 ml of 75 mg ml^-1^ saturated sodium carbonate, and its absorbance was measured at 760 nm after incubation for 2 h at room temperature in the dark. Quantifications were made based on the standard curve of gallic acid.

### Total Flavonoid Content

Total flavonoid content (TF) was measured by a modified colorimetric method (Huang et al., 2012). The appropriate dilutions of the extracted samples (1 ml) were mixed with 0.1 ml of 0.05 g ml^-1^ NaNO_2_. After 6 min, to each 0.1 ml of 0.1 g ml^-1^ AlCl_3_ solution was added; 5 min later, 1 ml of 1 mol L^-1^ NaOH was added to the reactive solution. This was then mixed well and allowed to stand for 15 min before measuring its absorbance at 510 nm. The TF was calculated and expressed as rutin equivalents.

### Proanthocyanidin Content

Proanthocyanidin content (PAC) of the blueberry extract was determined by the colorimetric assay developed by Price et al. (1978). Briefly, a 0.5% (w/v) solution of vanillin-HCl reagent (0.5% vanillin in 4% concentrated HCl, in methanol; 2.5 ml) was added to 0.5 ml of diluted extracts and standard (catechin) solutions, mixed thoroughly, and incubated at 30°C in the dark for 20 min. The absorbance of this solution was recorded at 500 nm against the corresponding blanks. The PAC was calculated and expressed as catechin equivalents.

### Total Anthocyanin Content

The blueberry extracts were filtered through a 0.22 μm filter (Millipore), and then injected to high performance liquid chromatography (HPLC) for analysis. This HPLC analysis was performed in an Aglient 1100 HPLC system (Agilent Technologies, USA) equipped with a binary pump and a diode-array detector (DAD). For the chromatographic analysis, a 250 mm × 4.6 mm, 5-μm particle size, end-capped reverse-phase Zorbax SB-C18 column was used (Agilent Technologies, USA). The running temperature was 35°C, with an injection volume of 10 μl. The detections were made at 520 nm, at a flow rate of 0.6 ml/min. Mobile phase A was a mixture of 6% HAc (ethanoic acid) and ultrapure water, whereas mobile phase B was a mixture of 6% HAc and acetonitrile. The gradient used was as follows: 5 to 10% B (from 0 to 5 min), 10 to 15% B (from 5 to 20 min), 15 to 20% B (from 20 to 35 min), 20 to 40% B (from 35 to 40 min), 40 to 80% B (from 40 to 45 min), 80 to 85% B (from 45 to 50 min), 85 to 5% B (from 50 to 55 min), and 5% B (from 55 to 60 min). To quantify anthocyanins, their peak areas were compared to the absorbance of a cyanidin 3-glucoside external standard (Sigma-Aldrich, Shanghai, China). Total anthocyanin content (hereon, ACs) was calculated as the sum of these peaks and expressed on a FW basis of fruit.

### E-Tongue Analysis

The overall taste profile of the blueberry was measured by an E-tongue (TS-SA402B, Intelligent Sensor Technology Inc., Kanagawa, Japan). This device consisted of an automatic sampler, a selective sensor array of six sensors (sourness, bitterness, astringency, umami, saltiness, richness, aftertaste-astringency, and aftertaste-bitterness), a signal acquisition instrument, and pattern recognition software. The surface of the selective sensors is combined with artificial lipid membranes has different response properties to chemicals on the basis of their taste. The selective sensors used in this study were: GL1 specific for sweetness, CA0 for sourness, C00 for bitterness and aftertaste-bitterness, AE1 for astringency and aftertaste-astringency, AAE for umami, CT0 for saltiness. The measurement principle of the E-tongue is based on the capability of tasty substances to change the potential of the sensors through electrostatic or hydrophobic interaction with the hydrophilic and hydrophobic groups of the lipid membranes. The sensors were dipped into the calibrated solution, which was 30 mmol L^-1^ potassium chloride and 0.3 mmol L^-1^ tartaric acid, then the electric potential measured for each sensor was defined as Vr. Then the sensors were dipped into the blueberry solution for 30 s, and the electric potential for each sensor was defined as Vs. The relative value (Rv) was represented by the differences between the potentials of blueberry sample and the reference solution (Vs-Vr). Then sensors were rinsed with fresh calibrated solution for 6 s and then dipped into the reference solution again. The new potential for the reference solution was defined as Vra. The difference Vra-Vr between the potentials of the calibrated solution after and before sample measurement is the “Change of Membrane Potential caused by Absorption value” (CPAv) and corresponds to the “aftertaste”. Before a new cycle measurement, sensors were rinsed for 90 s with a washing solution (30% ethanol) and then for 180 s with the calibrated solution. The “taste values” were calculated by multiplying the Rv and CPAv of the sensors for coefficients based on Weber-Fechner law, which gives the intensity of sensation considering the sensor properties for tastes (Kobayashi et al., 2010). For preparing the blueberry samples, the blueberry homogenate was first mixed with distilled water (1:2, g/ml) by vortexing. This mixture was then put into a 50 ml centrifuge tube and centrifuged at 5,000 rpm for 10 min. Next, the supernatant was filtered through three layers of gauze and the ensuing clear liquid was poured into the special beaker of the E-tongue, and analyzed at room temperature. In conducting this data collection sequence, it alternated between the tested calibrated solution and a given blueberry sample and repeated four times for both. The average of the last three sets of obtained data was used for the statistical analysis.

### Data Analysis

All data are expressed here as the mean ± SD (standard deviation). Statistical analysis of the above data relied on one-way analysis of variance (ANOVA), followed by Duncan’s multiple test, to identify significant differences among samples of blueberry grown at different locations, using SPSS v18.0 (SPSS Inc., Chicago, USA). An alpha level of *p* < 0.05 or less was considered to be significant.

Correlation analysis was performed among phytochemical conpounds and sensory properties using SPSS v18.0 (SPSS Inc., Chicago, USA). The correlation coefficients were analyzed by Pearson method, and significance was tested by two-tailed method. An alpha level of *p* < 0.05 or less was considered to be significant.

In order to test for similarities among blueberries sampled from different locations, principal component analysis (PCA) was applied to the data set. Locations were inserted in rows and response variables including phytochemical properties, and sensory values were placed in columns. The PCA analysis was implemented in the Origin2019 software (Massachusetts, USA).

## Results and Discussion

### Phytochemical Compounds of Blueberry Affected by Growing Locations

Significant differences in blueberry TSS, TA, and TSS/TA were found among the different growing locations (**Table 2**). The TSS among the 10 locations ranged from 9.4 to 14.2%, TA ranged from 0.33 to 0.55%, and TSS/TA ranged from 20 to 37.7. Blueberry grown in Shigu had the highest TSS compared with the other locations, having also the lowest TA content and highest TSS/TA. These results show that where blueberry is grown has a significant impact on its fruit berries’ TSS and TA, which is in line with other results (Correia et al., 2016).

**Table 2:** The content of total soluble solids (TSS), titratable acidity (TA), and TSS/TA in blueberry from different locations in China.

Location	TSS (%)	TA (%)	TSS/TA
CT^1^	10.3 ± 0.83de	0.52 ± 0.04ab	20.0 ± 1.84e
BM	12.1 ± 1.26b	0.55 ± 0.03a	22.0 ± 2.33de
DS	10.9 ± 0.73cd	0.39 ± 0.03e	28.3 ± 3.66bc
XW	10 ± 0.00de	0.33 ± 0.03f	30.7 ± 3.32b
XC	9.4 ± 0.88e	0.47 ± 0.04bc	20.2 ± 2.64e
SG	14.2 ± 1.27a	0.38 ± 0.04e	37.7 ± 5.75a
YQ	11.4 ± 0.77bc	0.42 ± 0.08de	28.3 ± 5.69bc
YS	10.6 ± 0.78cd	0.34 ± 0.02f	31.4 ± 2.78b
HK	10.1 ± 0.55de	0.49 ± 0.05bc	21.0 ± 2.03e
LP	11.4 ± 1.47bc	0.46 ± 0.03cd	25.1 ± 3.52cd

Values are the mean ± SD, n = 5; different letters within each column are significantly different at p < 0.05 according to Duncan’ s test.

^1^CT, Caotao town; BM, Baima town; DS, Dushan town; XW, Xuanwei town; XC, Xianchang town; SG, Shigu town; YQ, Yinqiao town; YS, Yousuo town; HK, Haikou town; LP, Longpeng town.

Growing location also had a significant effect on the TF, TP, PAC, and ACs of blueberry (**Table 3**). Across the 10 locations, the corresponding values for TF, TP, PAC, and ACs ranged from 0.94–2.07 mg rutin g^-1^ FW, 1.44–3.53 mg gallic acid g^-1^ FW, 2.40–9.53 mg catechin g^-1^ FW, and 0.26–1.34 mg Cyanidin-3-glucoside g^-1^ FW. Blueberry grown in Caota and Baima towns had the lowest TF, TP, PAC, and ACs content, whereas that grown in Shigu, Yinqiao, Yousuo, and Longpeng towns had the highest TF content, while Yinqiao and Longpeng towns had the highest TP content and Longpeng town had the highest PAC and ACs contents. Significant correlations among blueberry’s TF, TP, PAC, and ACs content were found in the samples whose contents were significantly positively correlated with the altitude of their growing locations (**Table 4**). Studies involving altitude have provided contradictory results for the phenols content of both blueberries and bilberries (Martz et al., 2010; Zoratti et al., 2015). Some researchers also found no clear relationships, or negative ones, between altitude and the ACs content of blueberries (Åkerström et al., 2010; Martz et al., 2010). In later work, Wang et al. (2015) and Zoratti et al. (2015) reported the ACs in blueberries and bilberries increased when grown at higher altitudes. In our study, the results suggested the TF, TP, PAC, and ACs content of blueberry increased with altitudes rising from 37 to 2,010 m. This could be due to the plant’s protective mechanisms against the stronger solar radiation and ultraviolet light generally prominent in high altitude regions (Jaakola et al., 2004; Zoratti et al., 2015). Such strong radiation and ultraviolet light could increase free radicals, such as reactive oxygen species (ROS), which activate the biosynthesis of flavonoids, especially anthocyanins to protect plant organs (Dudareva et al., 2020).

**Table 3:** The content of total flavonoids (TF), phenols (TP), proanthocyanidin (PAC), and anthocyanins (ACs) in blueberry from different locations in China.

Location	TF (mg rutin g^-1^)	TP (mg gallic acid g^-1^)	PAC (mg catechin g^-1^)	ACs (mg cyanidin 3-glucoside kg^-1^)
CT^1^	0.94 ± 0.19d	1.44 ± 0.24e	2.40 ± 0.63f	0.26 ± 0.07g
BM	1.11 ± 0.14cd	1.67 ± 0.25de	3.37 ± 0.89ef	0.40 ± 0.12fg
DS	1.24 ± 0.22c	1.97 ± 0.36d	4.47 ± 1.07e	0.59 ± 0.17de
XW	1.56 ± 0.20b	2.440.34c	6.10 ± 1.21cd	0.83 ± 0.18bc
XC	1.26 ± 0.08c	1.98 ± 0.17d	3.48 ± 0.41ef	0.45 ± 0.07ef
SG	1.96 ± 0.34a	3.07 ± 0.60b	7.07 ± 1.91bd	0.90 ± 0.20bc
YQ	2.06 ± 0.23a	3.29 ± 0.49ab	7.21 ± 0.92bc	0.93 ± 0.09b
YS	2.07 ± 0.21a	3.10 ± 0.43b	7.67 ± 1.36b	1.02 ± 0.18b
HK	1.65 ± 0.13b	2.89 ± 0.38b	5.88 ± 0.91d	0.74 ± 0.13cd
LP	2.06 ± 0.28a	3.53 ± 0.47a	9.53 ± 1.75a	1.34 ± 0.27a

Values are the mean ± SD, n = 5; different letters within a column are significantly different at p < 0.05 according to Duncan’s test.

^1^CT, Caotao town; BM, Baima town; DS, Dushan town; XW, Xuanwei town; XC, Xianchang town; SG, Shigu town; YQ, Yinqiao town; YS, Yousuo town; HK, Haikou town; LP, Longpeng town.

**Table 4:** Pearson correlation coefficients for altitude and phytochemical contents of blueberry.

	Altitude	TSS^1^	TA	TSS/TA	TF	TP	PAC	ACs
Altitude	1							
TSS	0.23^*^	1						
TA	-0.30^**^	-0.04	1					
TSS/TA	0.36^**^	0.60^**^	-0.80^**^	1				
TF	0.83^**^	0.28^*^	-0.48^**^	0.52^**^	1			
TP	0.83^**^	0.26^*^	-0.37^**^	0.43^**^	0.95^**^	1		
PAC	0.73^**^	0.28^**^	-0.45^**^	0.50^**^	0.91^**^	0.96^**^	1	
ACs	0.70^**^	0.26^*^	-0.45^**^	0.47^**^	0.88^**^	0.93^**^	0.99^**^	1

The * and ** indicate significance at p < 0.10 and p < 0.05, respectively.

^1^TSS, total soluble solids; TA, titratable acidity; TSS/TA, the ratio between TSS and TA; TF, total flavonoids; TP, total phenols; PAC, proanthocyanidin; ACs, anthocyanins.

The TSS and TA significantly increased and decreased with altitude, respectively, meanwhile the TSS/TA was increased with the rising altitude (**Table 4**). This suggested blueberry grown at high altitude could be sweeter, since the TSS/TA is considered as a rough but reliable indicator of fruit sweetness and is recognized as a key factor contributing to the flavor of blueberries (Almutairi et al., 2017). Our results agree with reports on mandarin (Rokaya et al., 2016) and apricots (Naryal et al., 2019); however, Correia et al. (2016) reported that altitude did not affect the TSS in blueberry, and an inverse relation was observed in bilberry and pomegranate, in that a higher TSS content predominated at lower altitudes (Mikulic-Petkovsek et al., 2015; Mphahlele et al., 2016). From our results, we infer that high altitude climatic conditions are favorable for blueberry’s accumulation of sugar contents. Sunlight at high altitudes is of higher intensity than at lower altitudes, contributing to an accelerated photosynthetic rate and metabolite accumulation (Naryal et al., 2019). Another factor that could increase TSS content arises from reduced respiration caused by the low temperatures characterizing high altitude zones, which would promote the synthesis and accumulation of carbohydrates in fruits (MacKenzie et al., 2011).

### Correlations Among Phytochemical Compounds of Blueberry

Significant correlations were also found between phenolic compounds and TAA, TA, and SS/TA (**Table 4**). These results were similar to findings from other studies, mainly on grape (Matsumoto et al., 2007) and pomegranate (Fawole and Opara, 2013). It may be that blueberry with high TSS are better able to sustain a high content of phenolic compounds, since physiologically they both increase simultaneously during the maturity stage of berries (Ribera et al., 2010).

### Correlations Between Sensory Properties and Phytochemical Compounds of Blueberry

**Figure 2:**
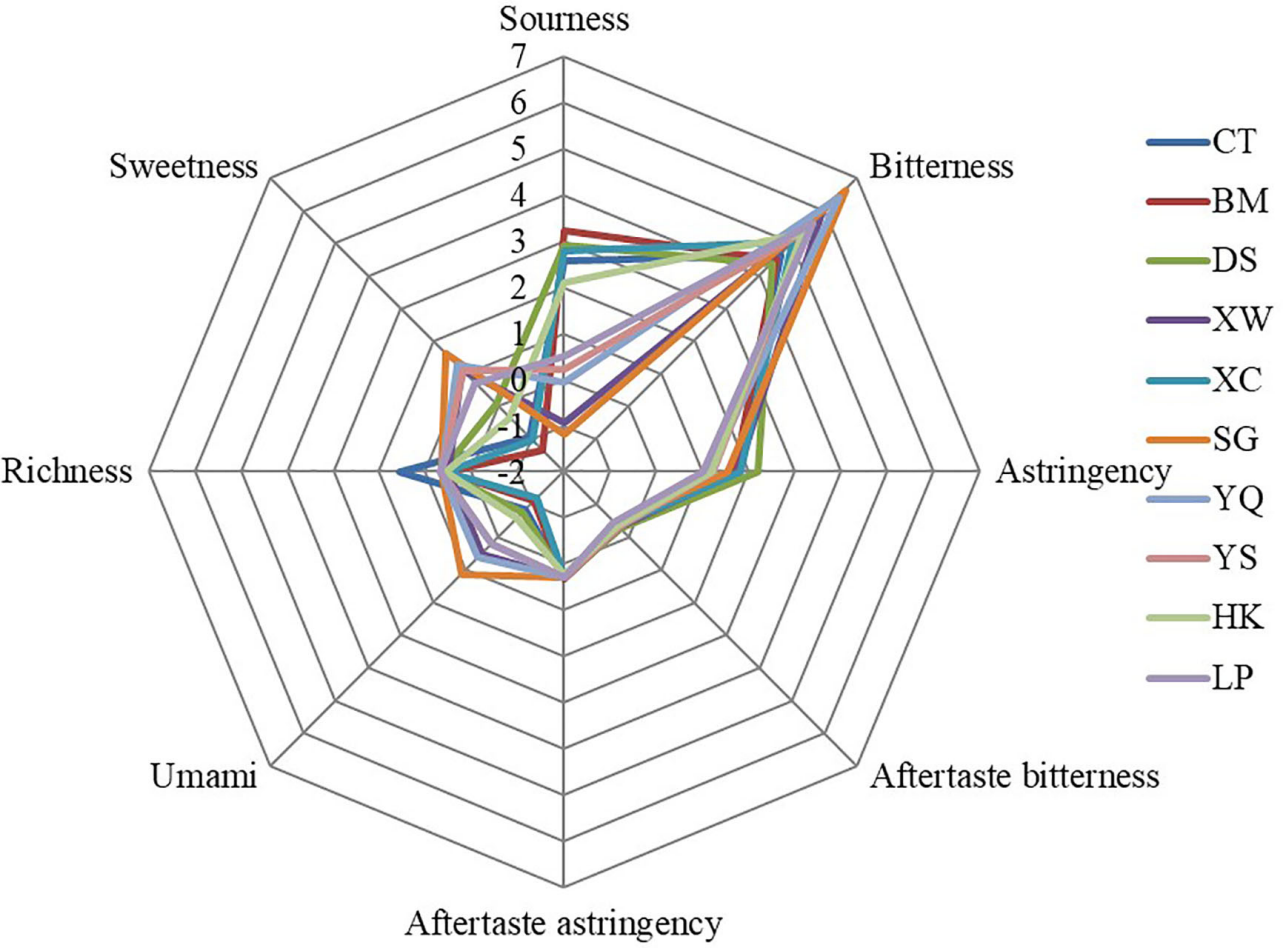
Spider plot for sensory scores of blueberry sampled from different locations in China. CT, Caotao town; BM, Baima town; DS, Dushan town; XW, Xuanwei town; XC, Xianchang town; SG, Shigu town; YQ, Yinqiao town; YS, Yousuo town; HK, Haikou town; LP, Longpeng town.

Significant differences were found among blueberry sensory scorings evaluated by the E-tongue among the 10 sampled locations (**Figure 2**). Furthermore, significant negative correlations were evident between altitude and sourness, astringency, aftertaste-bitterness, and richness, while altitude was significantly positively correlated with bitterness, umami, and sweetness of the berries (**Table 5**). Additionally, TSS was significantly positively correlated with bitterness, aftertaste astringency as well as umami and sweetness, yet negative correlated with sourness. The TA was also correlated with sensory scores but its trend was the opposite of that shown by TSS. The TF, TP, PAC, and ACs all had a similar association with the sensory values as found for TSS, which was positively correlated with bitterness, umami, and sweetness, and negatively correlated with sourness, astringency, aftertaste-bitter, and richness of the blueberries. Unlike the findings of Drewnowski and Gomez-Carneros (2000), we found that a high concentration of anthocyanin and total phenolics increased the astringent tastes of blueberry, a result similar to that reported by Bett-Garber et al. (2015), who studied the relationship between physicochemical contents and sensory values of six blueberry cultivars, and found that a sweet taste was positively correlated with the TSS and anthocyanin content of blueberry, while its sour taste was positively correlated with its TA.

**Table 5:** Pearson correlation coefficients between altitude, phytochemical properties, and sensory scorings of blueberry.

	Sourness	Bitterness	Astringency	Aftertastebitter	Aftertaste astringency	Umami	Richness	Saltiness	Sweetness
Altitude	-0.57**	0.76**	-0.70**	-0.48**	-0.14	0.62**	-0.34**	-0.76**	0.51**
TSS^1^	-0.28**	0.37**	-0.14	-0.20	0.30**	0.31**	0.04	-0.26*	0.27**
TA	0.64**	-0.47**	-0.09	-0.02	-0.03	-0.63**	0.34**	0.34**	-0.70**
TSS/TA	-0.67**	0.58**	-0.02	-0.10	0.17	0.67**	-0.24*	-0.40**	0.69**
TF	-0.60**	0.66**	-0.62**	-0.30**	0.09	0.64**	-0.42**	-0.68**	0.61**
TP	-0.54**	0.62**	-0.66**	-0.34**	-0.01	0.59**	-0.42**	-0.71**	0.57**
PAC	-0.58**	0.57**	-0.60**	-0.30**	0.07	0.62**	-0.39**	-0.62**	0.62**
ACs	-0.56**	0.56**	-0.58**	-0.32**	0.03	0.60**	-0.36**	-0.59**	0.60**

The * and ** values indicate significance at p < 0.10 and p < 0.05, respectively.

^1^TSS, total soluble solids; TA, titratable acidity; TSS/TA, the ratio between TSS and TA; TF, total flavonoids; TP, total phenols; PAC, proanthocyanidin; ACs, anthocyanins.

**Figure 3:**
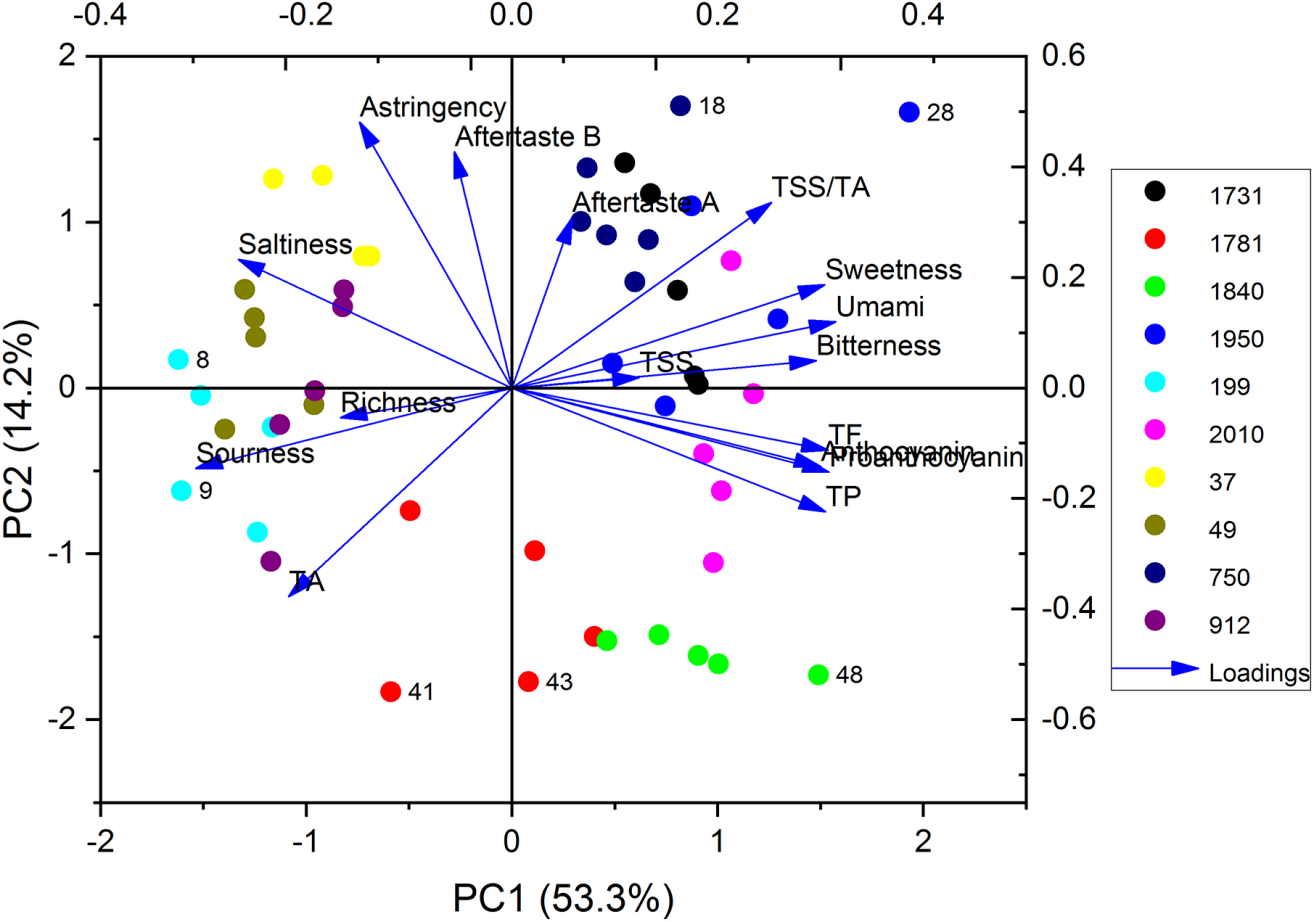
Principal component analysis (PCA) of phytochemical analyses and sensory scores of blueberry from different locations in China.

Sensory scorings and phytochemical analyses were also processed by PCA, for which the two-dimensional biplots of scores and loading of the different locations are presented in **Figure 3**. Principal component 1 (PC1) and PC2 respectively represented 53.3 and 14.2% of the total variance in the data set. The PC1 was dominated by sensory scores: umami, and sweetness, as well as phytochemical contents: TF, TP, PAC, and ACs. The PC2 was dominated by sensory scores: astringency, aftertaste-astringency and aftertaste-bitterness as well as some phytochemical content: TA and TSS/TA (**Figure 3**). The short distance among TF, TP, PAC, and ACs suggested they have a similar influence upon the evaluation of blueberry’s food quality. The PCA plot showed that blueberry from different locations could be effectively discriminated, although the values for some locations were clustered and showed some overlap (**Figure 3**). Blueberry plants grown at high altitudes were located on the right of the x-axis, which suggested their fruits had high sensory scores for umami and sweetness, and a high content of TF, TP, PAC, and ACs. These results indicated that for the production of high-quality berries with respect to their sweetness and ACs content, it would be better to established blueberry fields at high altitudes. However, it was hard to discriminate the  uality of blueberry from closing altitudes, due to the overlapping (**Figure 2B**). Therefore, looking ahead, more environmental factors should be considered, such as soil type and cultivation methods, since these two have an important role to play in determining blueberry properties and quality as food (Connor et al., 2002; Ehret et al., 2014; Zoratti et al., 2015).

## Conclusion

A systematic evaluation of various quality characteristics and sensory values of blueberry ‘Brightwell’ from 10 locations in China demonstrated that both sets were affected by their location, while blueberry sensory aspects were markedly influenced by co-occurring phytochemical contents. Bitterness, umami, and sweetness of berries were positively correlated with TSS, TF, TP, PAC, and ACs in blueberry; their sourness was positively correlated with TA but negatively correlated with TSS, TF, TP, PAC, and ACs. In any case, the sensory scores and values of TSS, TA, TF, TP, PAC, and ACs clearly depended on altitude. Taken together, we suggest that blueberry plants grown at high altitude are apt to yield high-quality berries with regard to their sweetness and ACs content.

## Data Availability Statement

The raw data supporting the conclusions of this article will be made available by the authors, without undue reservation.

## Author Contributions

WH and HY conceived, designed the experiments, and revised the manuscript. QZ, GD, LT, and HW performed the experiments. QZ and GD wrote the manuscript. YR, QZ, and GD analyzed the data. GT revised the manuscript and improved the language. All authors contributed to the article and approved the submitted version.

## Funding

This study was funded by the National Natural Science Foundation of China (Nos. 31301838, 31601709); the Natural Science Foundation of Jiangsu Province, China (Nos. BK20160597, BK20170615); and the Key Research and Development Plan (Modern Agriculture) of Jiangsu Province, China (No. BE2017373). 

## Conflict of Interest

The authors declare that the research was conducted in the absence of any commercial or financial relationships that could be construed as a potential conflict of interest.
